# The Impact of Three-Dimensional Printer Technology on the Accuracy of Dental Implant Models

**DOI:** 10.3390/ma18091902

**Published:** 2025-04-23

**Authors:** Alexander Strunz, Lara Berger, Anna Seidel, Johannes Ries, Werner Adler, Manfred Wichmann, Ragai Edward Matta

**Affiliations:** 1 Department of Prosthodontics, University Hospital Erlangen, Glückstrasse 11, 91054 Erlangen, Germany; alexander.strunz@uk-erlangen.de (A.S.); lara.berger@uk-erlangen.de (L.B.); anna.seidel@uk-erlangen.de (A.S.); johannes.ries@uk-erlangen.de (J.R.); claudia.ehrhardt@uk-erlangen.de (M.W.); 2 Institute of Medical Informatics, Biometry and Epidemiology (IMBE), Friedrich-Alexander-University Erlangen-Nuremberg, Waldstrasse 6, 91054 Erlangen, Germany; werner.adler@uk-erlangen.de

**Keywords:** computer-aided design/computer-aided manufacturing (CAD/CAM), digital workflow accuracy, implant dentistry, 3D printing in dentistry, additive manufacturing dentistry

## Abstract

This study examines the impact of different 3D printing technologies on the accuracy of implant positions in printed dental models, a crucial factor in implant-supported prosthetics. A standardized titanium model with three bone-level implants was scanned using an industrial scanner to create a virtual reference model. Ten intraoral scans of the same model were performed, and the generated STL files were used to design physical models printed with three different 3D printers: two utilizing digital light processing (DLP) technology and one employing stereolithography (SLA) (n = 30). The printed models were then rescanned, and deviations from the reference STL file were analyzed. Results showed that the SLA printer exhibited the highest deviations (0.26 ± 0.17 mm), whereas the DLP printers demonstrated greater accuracy, with one DLP system (0.07 ± 0.02 mm) performing slightly better than the other (0.12 ± 0.13 mm). The SLA printer exhibited the most significant errors in the vestibulo-oral and occlusal-apical directions. The findings suggest that DLP printers offer superior precision for implant-supported restorations in digital workflows. Clinically, the choice of 3D printing technology significantly impacts model accuracy, emphasizing the importance of selecting the appropriate printer based on the required precision.

## 1. Introduction

The treatment of patients with implant-supported or implant-retained solutions plays an increasingly important role in everyday clinical practice [1]. Dental implants offer advantages in terms of esthetics as well as tooth preservation and function. They are increasingly being used not only to replace individual lost teeth, but also to treat edentulous patients [2]. Implants are a valuable alternative to mucosa-supported full dentures in such situations [3].

After successful implant placement and a variable healing phase, the prosthetic restoration is fitted, involving impression taking, restoration fabrication, and placement on the patient [4]. Implant prosthetic restorations can follow conventional or digital workflows, which should not be viewed as strictly separate.

In the conventional workflow, open and closed impressions are used, with studies showing that the open approach is more precise [5,6]. After fabrication of plaster models from patient impressions, the superstructure can be constructed either conventionally or digitally after digitization [7].

Scanbody geometries are then superimposed on the virtual 3D impression to register the exact implant position [1,8,9]. Studies on intraoral scanners indicate that their precision is clinically acceptable for individual implants or short-span bridges, but not yet sufficient for long-span restorations, prompting the investigation of scanning aids for such cases [10,11,12,13]. The digital design of the superstructure is directly based on intraoral scan data, minimizing errors by reducing additional transmissions and manual steps [8].

However, in most cases, a physical model is required to check and finalize the machine-made superstructure. On the one hand, manual work steps such as individual veneering can only be carried out on a model. On the other hand, restorations fabricated in the laboratory are classified as medical devices according to European law and must be checked by the dental technician for criteria such as gap-free and rocking-free fit on the model, correct clearance, positioning, or proximal contact verification before delivery to the dentist [14,15,16].

As the digital workflow becomes increasingly established, there is a need to transfer the information obtained during intraoral scanning into an exact physical model. In addition to subtractive methods, in which the models are milled from various plastics using milling machines, additive methods are also available [17,18].

Various 3D printing systems have increasingly found their way into everyday dental practice and dental technology in recent years. Initially used primarily for the fabrication of models, the technology now enables the precise manufacturing of provisional or definitive prostheses, bridges, or crowns [19,20]. Additionally, 3D printing is employed in the precise planning of surgical procedures, such as the creation of drill guides for implant placements [21,22]. Frequently used technologies include digital light processing (DLP) and stereolithography (SLA) methods [17,19,20]. The main advantages over subtractive production methods are their material efficiency and speed, which also result in lower costs [21,22]. Studies investigating the accuracy of 3D-printed models show that clinically acceptable results are achieved [23,24]. At the same time, numerous factors were identified that significantly influence the results of an additively manufactured model. In addition to the printer technology, the specific printer model, and the material used, parameters such as build angle or layer thickness can also have a significant impact on quality [19,25,26].

Stereolithography (SLA) was the first 3D printing process and served as the foundation for all subsequent technologies. It remains widely used today. During the printing process, the build platform is submerged in a reservoir filled with printer resin until it stops just before reaching the bottom. A UV laser is then used to polymerize the first layer of the object onto the build platform through the translucent window of the reservoir. The build platform subsequently moves upward allowing fresh resin to flow beneath it before returning to the viewing window, where the next layer is polymerized [27,28,29]. Digital light processing (DLP) follows a similar principle to SLA, with the key distinction being the light source used for photopolymerization. Instead of a laser, DLP employs a semiconductor plate with microscopically small mirrors, known as a digital micromirror device, which exposes the entire cross-section of the object at once [19,28,29].

Ultimately, many processes in additive manufacturing with 3D printers are specially tailored to the printer model being used. For example, the resolution of different printers varies and material properties such as the viscosity of the printer resin or the wavelength and light intensity required for curing must be compatible between the printer and its resin [19]. While both mechanical and biological material properties are of central importance for applications such as drilling templates or temporary and permanent restorations, it is above all the precision of the material that is decisive for models that are used purely in the dental laboratory [24,25,26]. Table 1 shows additional information on the printer resins used and their recommended processing parameters.

While the influence of individual factors on the quality of printed models has been well researched [25,26,27,28], the extent to which individual printers and their technologies, together with the recommended materials are suitable for the production of high-precision implant models has been little investigated to date. The aim of this study was therefore to compare the achievable accuracy of different validated systems for the fabrication of implant models. The data presented in the study by Revilla-León et al. [24], which examined the accuracy of 3D-printed compared to conventionally produced implant models, has already demonstrated that 3D-printed models can be used in a comparable manner to the conventional workflow. Building on this, the novelty of the present study lies in the comparative analysis of different self-contained workflows for a comprehensive evaluation of these systems and their suitability for the production of implant models. To the best of our knowledge, there is no available information regarding the positioning accuracy of laboratory analogs in comparison to a reference for the printers examined in this study, using the printer resins employed. The influence of the printer model and its technology was investigated using the parameters, materials, and post-processing specified by the manufacturer of each printer.

The null hypothesis is that the printer model and its technology have no influence on the position of the implants in the printed implant model.

## 2. Materials and Methods

The aim of this in vitro study was to examine the trueness of the implant position in printed models as a function of the printer technology used. The accuracy was investigated as a function of three different 3D printers using a total of two different technologies. The ASIGA Max UV (ASIGA, Sydney, Australia) and the Straumann P30+ (2) printer (Rapidshape, Heimsheim, Germany) were examined as representatives of DLP technology, while the SLA printer Form 3+ (3) (Formlabs GmbH, Berlin, Germany) was compared.

**Figure 1:**
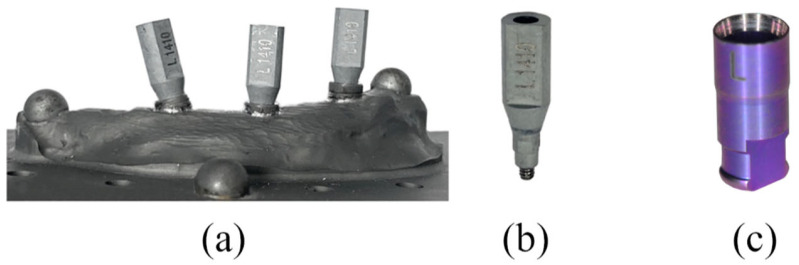
(**a**) Medentika scanbodies screwed onto reference titanium model; (**b**) Medentika Scanbody L-Series L1410; (**c**) laboratory analog from Medentika (L-Series L81; Straumann GmbH, Freiburg im Breisgau, Germany).

As a reference model, an edentulous maxilla was milled from titanium using a CNC milling machine (NobelProcera, Nobel Biocare Services AG, Zürich, Switzerland). The reference model represented the left half of the upper jaw. The total volume was 7269 mm^3^ with a surface area of 4422 mm^2^. Three bone-level implants (TiZr, ø 4.8 mm RC, SLA 12 mm, Roxolid, Loxim; Straumann AG, Basel, Switzerland) were then placed in the milled titanium model and fixed using a laser. The implants in the anterior and premolar regions were placed at a right angle to the model base and the implant in the posterior region was placed at an angle of approximately 15° to the distal (see Figure 1a). Scanbodies from Medentika (L-Series L1410; Medentika GmbH—Straumann Group, Hügelsheim, Germany) were selected to record the implant position according to a digital workflow (see Figure 1b).

In order to ensure a non-reflective, matte surface, the titanium model was sandblasted with aluminum oxide abrasive, leaving out the implants. To increase the accuracy of the individual scan alignments, the model was then covered with measurement markers (Reference markers 0.8 mm; GOM GmbH, Braunschweig, Germany). The titanium model with manually screwed-in scanbodies was digitized using the ATOS So4 II industrial scanner (ATOS, GOM GmbH, Braunschweig, Germany) to generate a Standard Tessellation Language (STL) reference file [30].

Using the Primescan intraoral scanner (Dentsply Sirona, Bensheim, Germany; Primescan AC; Connect SW 5.2), 10 scans of the same model were then taken. To simulate as realistic a patient scenario as possible, the scanbodies were screwed in manually before each scan. The generated intraoral scan STL files were imported into the CAD software 3Shape (3Shape Dental Manager, Copenhagen K, Denmark, Version 2.21.1.0) and models were constructed. The implant position was aligned using a 3-point overlay process. As in other studies, this method showed better results than automatic single-point alignment [30]. The implant-analog-holder offset (IAH), which refers to the gap between the laboratory analog and the printed model, was set to 0.06 mm, as other studies have shown that the highest positional accuracy is achieved for IAH offsets between 0.04 and 0.06 mm [31].

**Figure 2:**
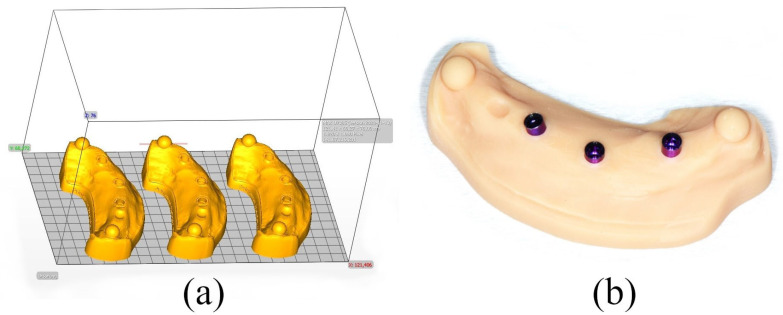
(**a**) Nesting of digital implant models using ASIGA Composer nesting software as an example; (**b**) printed model after post-processing and with laboratory analogs placed.

The STL model data (n = 10) were then exported and converted into 10 physical models each (n = 30) using three different 3D printers. The first of the 3D printers with DLP technology to be tested was the ASIGA MAX UV (group A) (ASIGA, Sydney, Australia). After the nesting process with the associated nesting software ASIGA Composer (Version 1.2.12), 10 models were produced with the printer resin IMPRIMO^®^ LC Model Beige (SCHEU-DENTAL GmbH, Iserlohn, Germany) (n = 10). In addition, the DLP printer Straumann P30+ (group B) (Rapidshape, Heimsheim, Germany) was examined, using the nesting software AUTODESK NETFABB (Autodesk, Version 2022.0) and the printer resin PRO RESIN, MODEL X (Straumann GmbH, Freiburg im Breisgau, Germany). The third system examined was the SLA printer Form 3+ (group C) (Formlabs GmbH, Berlin, Germany) with the nesting software PreForm (Version 3.33.1) and the printer resin Formlabs Dental Model Resin (Formlabs GmbH, Berlin, Germany). The selection of materials and parameters for the printing process was based on the manufacturers’ recommended specifications for the 3D printers. However, all models were nested with their base parallel to the build platform, and a layer thickness of 50 µm was maintained (see Figure 2a). Table 1 provides more detailed information on the materials used and their corresponding processing parameters.

The post-processing of the printed models (n = 30) was also carried out according to the manufacturer’s specifications using the washing and light-curing devices associated with the printers. All models printed with DLP technology were post-processed with the Straumann P-Wash and P-Cure device (Straumann GmbH, Freiburg im Breisgau, Germany). The parameters stored by the manufacturer were selected for the corresponding materials. The P-Wash unit uses two separate containers of isopropanol as a solvent, one for an initial rough cleaning and a second pass for final cleaning. Post-curing in the P-Cure unit takes place under vacuum. For the SLA-fabricated models, cleaning was performed using the Form Wash (Formlabs GmbH, Berlin, Germany) cleaning device. The models were washed in isopropanol for 10 min before being post-cured for 30 min in the Form Cure light-curing unit.

After post-processing, the printed models were fitted with laboratory analogs from Medentika (L-Series L81; Straumann GmbH, Freiburg im Breisgau, Germany) (see Figure 1c and Figure 2b).

The models were then coated using an airbrush gun and a solution of Rutile Titanium White in ethanol (GOM GmbH, Braunschweig, Germany) to avoid reflections and covered with reference markers. The scanbodies were then screwed into the laboratory analogs of the printed models and the models were digitized using the ATOS So4 II industrial scanner (ATOS, GOM GmbH, Braunschweig, Germany) (n = 30). The digitization of the printed models was performed for all groups within 2 days after their production in order to minimize dimensional changes due to longer storage periods.

**Figure 3:**
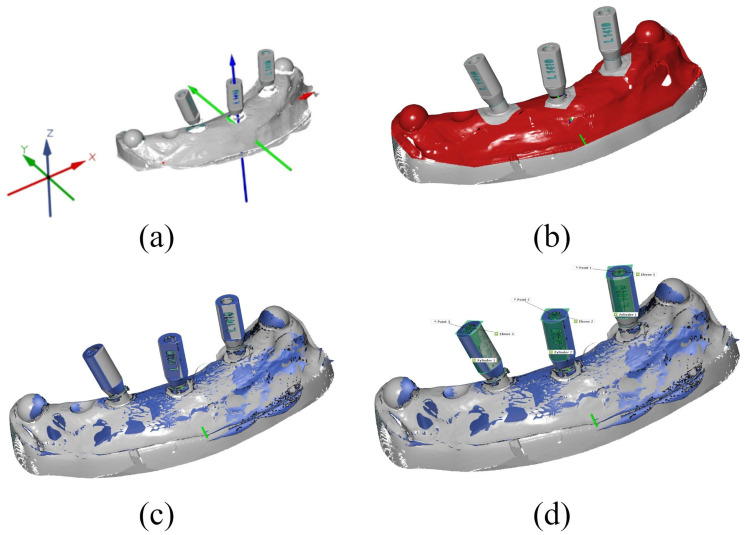
(**a**) The 3D coordinate system used, where the X-axis represents the mesio-distal orientation, the Y-axis represents the oro-vestibular orientation, and the Z-axis represents the cranial-caudal orientation. (**b**–**d**) Examples of the virtual calculation of 3D deviations in the ATOS Professional Software: (**b**) surface selection of the digitized printed models for the matching process with the reference STL data set, (**c**) overlaying of the STL data with the reference STL file, and (**d**) determination of the deviation between the STL of a printed model and the reference STL using a fitting cylinder and fitting plane.

The accuracy of the implant positions in the printed models was analyzed by superimposing the resulting STL data with the reference STL file in the ATOS Professional software (v2018; GOM GmbH, Braunschweig, Germany) using a best-fit alignment of the model surface while omitting the scanbody surface (see Figure 3b–d). This step is essential to prevent alignment with the scanbody, as this would be undesirable at this stage. A best-fit alignment that includes the scanbody surface would distort the results, leading to significantly reduced deviations.

In order to compare the implant position with the reference STL file, measurement points were determined on the scanbodies of the reference STL and the individual STL data sets. For this purpose, a fitting cylinder with maximum contact to the scanbody surface and a maximum congruent plane perpendicular to the fitting cylinder was constructed. The measurement point for each implant is defined by the intersection of the cylinder axis with the vertical plane at the top of each scanbody, resulting in three measuring points for each model. Deviations from these reference points were plotted in a 3D coordinate system, with the X-axis representing the mesio-distal orientation, the Y-axis representing the oro-vestibular orientation, and the Z-axis representing the cranial-caudal orientation (see Figure 3a). In addition to the deviations in the individual planes, the Euclidean distance dXYZ was determined as the 3D deviation resulting from the vector of the displacement relative to the three spatial planes. The deviations from the reference STL were given as maximum, average, and minimum deviation in mm (see Table 1). This method can be classified as suitable, as it has also been used successfully in other studies [6,30,32,33].

**Figure 4:**
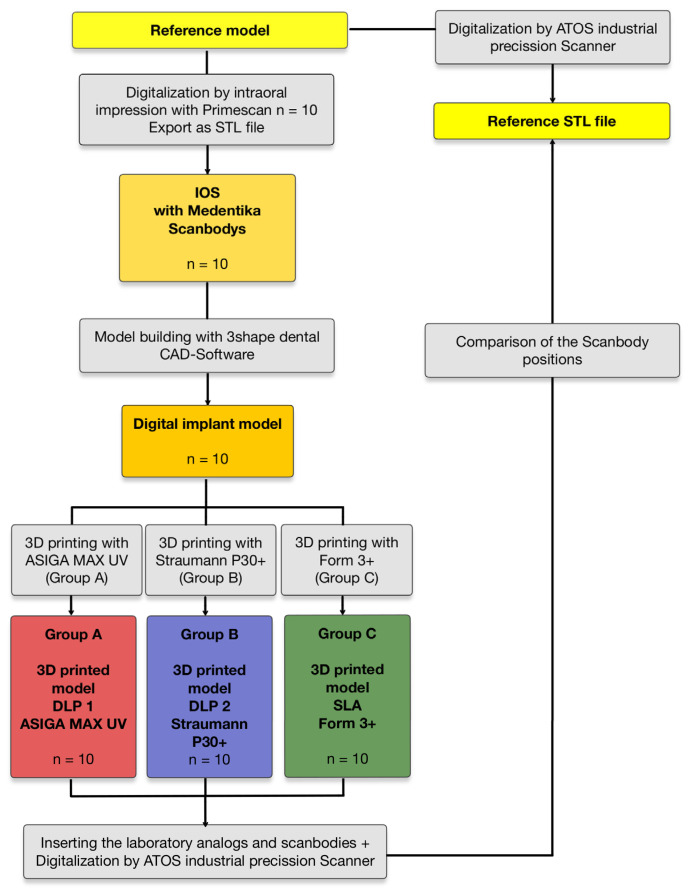
A representation of the study procedure with the individual study groups.

The data were categorized into three groups: group A includes measurements from models printed with ASIGA MAX UV, group B contains those from Straumann P30+, and group C consists of models produced with Form 3+. Figure 4 provides an overview of the study design.

Statistical analysis was performed with the software R (Version 3.1.1 2014-07-11, R Core Team, R Foundation for Statistical Computing, Vienna, Austria). The Mann–Whitney U test was used to compare the printers with each other. This test is particularly suitable if the data are not normally distributed or correspond to ordinal scales, as it makes no assumptions about the distribution of the data. The significance level was set at *p* < 0.05, which means that the probability of observing an outcome that is at least as extreme as the observed outcome must be less than 5% for a true null finding to be considered statistically significant. This strict threshold (*p* < 0.05) was chosen to ensure high precision in the results and to minimize the risk of 1st type errors (falsely rejecting the null hypothesis).

## 3. Results

Overall, the data obtained showed that the implant models produced by the SLA printer (Form 3+/Formlabs/SLA) (group C) exhibited significantly greater discrepancies (0.26 ± 0.17 mm) in the overall three-dimensional deviation of the implant positions, represented by the Euclidean distance dXYZ, compared to the reference, than the models produced by the two printers using DLP technology (*p* < 0.001) (see Table 2).

**Figure 5:**
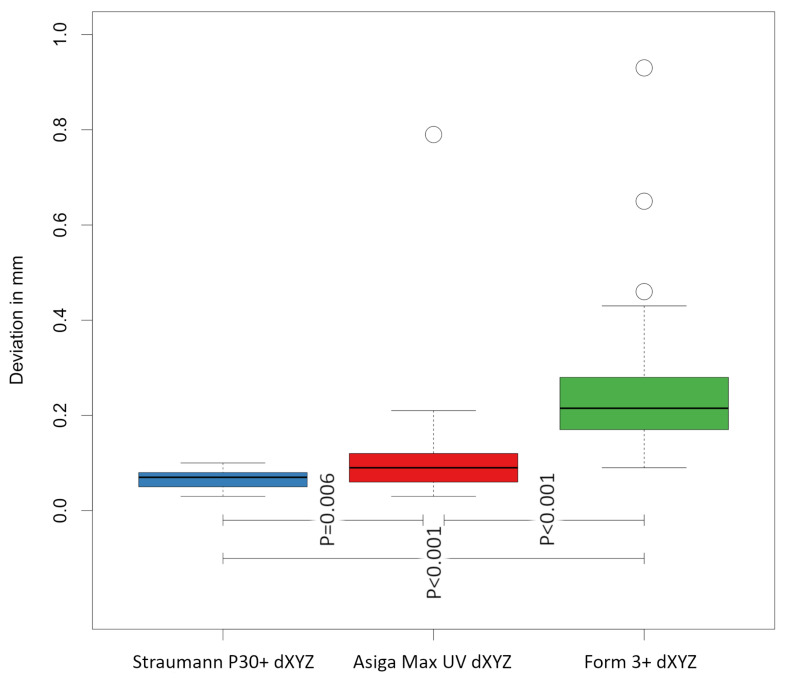
Statistical comparison of groups A and B, B and C, and A and C, with corresponding *p*-values for Euclidean distance dXYZ. The white circles represent the outliers.

**Figure 6:**
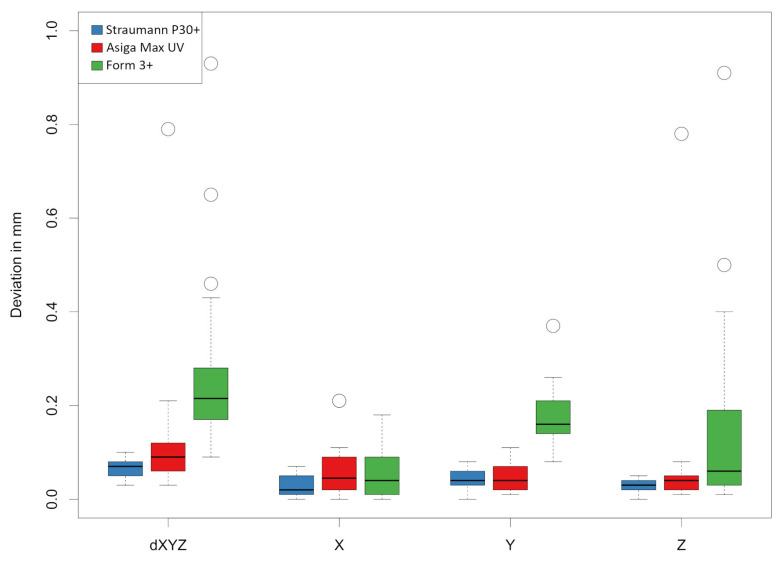
Boxplots for the comparison of groups A, B, and C for the Euclidean distance dXYZ, as well as the individual spatial planes X-, Y-, and Z-axis. The white circles represent the outliers.

The two DLP printers again showed comparable values to each other, whereby the Straumann P30+ printer (0.07 ± 0.02 mm) (group B) achieved significantly higher accuracy in the overall deviation compared to ASIGA MAX UV (0.12 ± 0.13 mm) (group A) (*p* = 0.006) (see Figure 5). The collected data show a significant standard deviation, particularly in the Z-axis measurements of the SLA printer Form 3+. A lower standard deviation is observed in the measurements of the DLP printer ASIGA MAX UV, with the lowest standard deviations found in the DLP printer Straumann P30+. Our research has shown that the repeatability of the examined Straumann P30 DLP printer can be classified as very good. No outliers were observed, unlike the case with the DLP printer ASIGA MAX UV, and to an even greater extent with the SLA printer Form 3+. Figure 5 and Figure 6 illustrate these outliers as circles on the corresponding measured values.

When looking at the deviations in the individual spatial planes, the models manufactured using SLA technology (group C) show a greater deviation in the Y-axis (0.17 ± 0.06 mm) and Z-axis (0.14 ± 0.20 mm) compared to the X-axis (0.05 ± 0.05 mm) (see Figure 6).

No significant difference was found with regard to the implant position in the anterior, premolar, or posterior region.

## 4. Discussion

The digital workflow is increasingly being used in the treatment of patients with fixed dentures on implants. The focus is on saving time for both practitioners and patients, reducing costs and material usage, and minimizing sources of error [7,8,14].

In addition to the long-established subtractive methods of manufacturing dentures using CNC milling, additive manufacturing methods using 3D printers are becoming more relevant. The production of models is a frequent application of this technology [19,20,21,22].

If models are designed and fabricated purely based on an intraoral scan, there is no physical working basis for the manual post-processing and subsequent final inspection of implant restorations fabricated in the CAD/CAM workflow [6,7,8]. This makes it necessary to transfer the information obtained from the intraoral scan as accurately as possible into a physical model [14].

For this study, three different printers were selected for the production of implant models. Two printers using DLP technology were compared with each other as well as with a printer using SLA technology. The high-precision industrial scanner ATOS So4 II was used for this study both to generate a reference STL and for the 3D evaluation of the printed models, which also allows potential errors in the intraoral scan to be detected [6,32,33]. To minimize other factors influencing the printed models, the same parameters were applied to all models and the manufacturer’s validated printer resin was used.

Numerous studies have examined the quality of printed models [27,28,29,34,35,36,37,38]. Various factors were found to influence the accuracy of such models. In addition to the printing technology examined in this study, the selected printing parameters, such as the build angle, the layer thickness, placement on the build platform, and printing temperature, also play a role [25,27,34,35]. The procurement of the printing resin and the post-processing after the printing process also have an influence on the quality [36]. The influence of different printers and their technology on the positioning accuracy of the implant analogs in the model is still poorly understood. In this context, the aim of this study was to determine whether clinically acceptable implant models can be produced with the selected printers and which systems produce the best results with the processes specified by the manufacturer.

**Figure 7:**
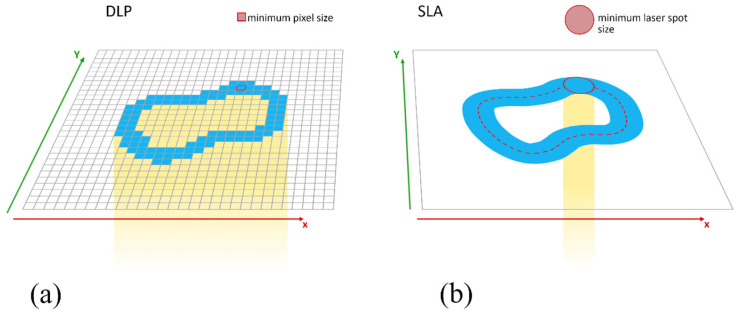
A comparison of the functionality of DLP versus SLA in relation to the x/y-axis and the resulting resolution. (**a**) In the DLP process, the entire layer is exposed simultaneously. The resolution is determined by the pixel size. (**b**) In the SLA process, the layer is drawn by a laser dot. The resolution depends on the diameter of the laser spot.

To test the null hypothesis, the deviation of the implant position was compared by superimposing the scanbody geometry in the printed model with the reference model, with regard to the Euclidean distance and along the x, y, and z axes. The x and y axes together ensure two-dimensional movement in the print plane and thus generate the horizontal outline of the model to be printed. The resolution of the printer plays a decisive role here. In the case of DLP technology, this depends on the pixel density of the projector. In this context, a smaller print area also means a higher resolution. With SLA technology, this resolution is theoretically infinitely variable, as the laser has no fixed pixels. In practice, the resolution is limited by the dot size of the laser and its movement via the mirror system [29]. Figure 7 shows a schematic representation of the different operating modes in relation to their resolution. The z-axis adds the third dimension by changing the height of the print. The precision of the movement of the building platform and the curing depth of the light source used for polymerization may play a decisive role here. The larger deviations in the z-axis shown in our study for the SLA printer compared to the two DLP printers could indicate a lower precision in this movement.

Overall, the null hypothesis was rejected, as there were statistically significant deviations in the implant positions in the printed models depending on the printer used. The lower deviations in Euclidean distance achieved by the two DLP printers (0.07 ± 0.02 mm for Straumann P30 group B and 0.12 ± 0.13 mm for ASIGA MAX UV group A) can be compared with values from similar studies [27,28,37]. In a study in which surgical guides were produced using DLP printers (D20 II; Rapid Shape) and SLA printers (Form 2; Formlabs), Vinzenz Le et al. found that the accuracy of the DLP-produced guides was higher [28]. Elisa Caussin et al. explained in a systematic review that for small print volumes, DLP printers enable higher accuracy than SLA printers, as the resolution of the DLP technology’s projector is higher on a smaller area than can be achieved by the laser of an SLA printer [29]. At 85 μm, the diameter of the laser spot on the SLA Form 3+ printer examined in this study is larger than the individual pixels of the DLP printers examined. According to the manufacturer, these have a resolution of 62 µm (ASIGA MAX UV) and 34 µm (Straumann P30). The resolution of the printers examined in the x and y planes thus follows the same gradation as the results measured in this study with regard to the accuracy achieved. A direct correlation with the resolution of the printer used is therefore very likely.

A study that examined two of the printers investigated here with regard to the accuracy of a printed maxillary dental arch also found a higher trueness for the DLP printer ASIGA Max UV compared to the SLA printer Form 3+. The simultaneous and therefore more even polymerization in each layer was cited as a possible reason for this [27]. In a systematic review from 2020, deviations of 59-150 µm were classified as clinically acceptable for implant prosthetic restorations on printed models [38]. In the present study, all measured deviations of the DLP printer Straumann P30 (group B) showed values below 150 µm, so that the printer can be assessed as suitable for the production of implant models. On average, the measured values of the DLP printer ASIGA Max UV (group A) were also below 150 µm, although a deviation above 150 µm was measured at 3 of the 30 measuring points. With a maximum deviation of 150 µm, the models of the SLA printer (group C) can be rated as clinically unacceptable, which showed significantly larger deviations of 0.26 ± 0.17 mm both in individual measurements and on average. The largest deviations were found in the Y (0.17 ± 0.06 mm) and Z axes (0.14 ± 0.20 mm). In a previous study in which the positional accuracy of laboratory analogs in models produced with different printer technologies was also investigated, a larger overall deviation was found for the SLA printer compared to the DLP printer investigated, whereby higher accuracy in the Z-axis was found for the SLA printer than for the DLP printer [24]. However, the comparability of these data with the present study is limited by the different printers and printing parameters used.

The various factors already identified, such as the printer resin and the post-processing used, which make it difficult to draw clear conclusions about the sole dependence of trueness on the printer used and its technology, can be cited as a limitation of this study. Furthermore, all data collected in this study were based on intraoral scans in an idealized in vitro situation. It is to be expected that patient-specific factors in the clinical setting generate additional errors that could accumulate in the further workflow. As a potentially relevant parameter in the design of the models in the CAD software, the set implant-analog-holder (IAH) should be considered, which could influence the positioning of the analogs in the printed model. In the present work, a spacer of 0.060 mm was selected, as this proved best results in preceding studies and was the recommendation of the model design software [31]. The extent to which different values influence the position of the analog should be investigated further. In order to produce the most accurate results possible, a calibration was carried out for each printer before the printing process according to the manufacturer’s specifications. A reduced calibration interval or irregular maintenance of the 3D printers could also have a negative impact on the trueness of the printed models.

Future studies should extend the results obtained here with other printers and materials and add clinical parameters such as fit, occlusal, and proximal contacts of implant prosthetic restorations fabricated with printed models. In addition, in view of the large number of different laboratory analogs for printed models, the question arises as to what influence their technology and geometry have on the positional accuracy in the printed model. While the results presented here focus on the dental technology aspect of the digital implant workflow, it still allows conclusions to be drawn about, for example, the suitability of different printer technologies for the production of surgical guides. It is recommended that future studies investigate the extent to which sufficiently precise surgical guides can be produced with different systems and which manufacturing parameters have an influence on accuracy.

## 5. Conclusions

Within the limitations of this in vitro study, the following conclusions were drawn:

1. The technology of the printer used has a significant influence on the accuracy of 3D-printed implant models (*p* < 0.001).
2. The two DLP printers examined show mostly higher trueness, (0.07 ± 0.02 mm) and (0.12 ± 0.13 mm), respectively, than the SLA printer examined (0.26 ± 0.17 mm).
3. The DLP printer P30 shows very good reproducibility in its results and can be recommended for the fabrication of implant models.

Based on this study, the printer used should be carefully selected depending on the accuracy required for the respective indication. For the production of implant models, a high-precision dental DLP printer is recommended.

It is to be expected that newer generations of 3D printers will increase their accuracy. Larger build platforms and a trend towards the automation of individual work steps, such as separation from the build platform, will make production more efficient and further reduce potential sources of error. The obtained results also provide insights into the suitability of the employed printer technologies for additive manufacturing of other materials, such as ceramics, within a digital workflow. High-precision 3D printers could be utilized for the fabrication of patient-specific ceramic implant bodies, thus expanding the range of applications for 3D printing in implant dentistry.

## Figures and Tables

**Table 1:** More details on the printer resins and their recommended processing parameters.

	Resin 1 IMPRIMO^®^ LC Model Beige (DLP—ASIGA MAX UV)	Resin 2 PRO RESIN, MODEL X (DLP—Straumann P30)	Resin 3 Formlabs Dental Model Resin (SLA—Form 3+)
composition	alkoxylated bisphenol-A dimethacrylate, phosphine oxide, stabilizers, dyes, and pigments	UDMA, diacrylate, acrylic resin, phosphine oxide	bismethacrylate, methacrylate monomers, ethyl phenylphosphinate
layer height	50 µm	50 µm	50 µm
build angle	0°	0°	0°
mean printing time	37 min 32 s	45 min 12 s	1 h 50 min 8 s
printing temperature	30.0 °C	35.0 °C	35.0 °C
wavelength	385 nm	385 nm	405 nm
light intensity	11.7 $\frac{mW}{{cm}^{2}}$	no available information	250 mW Laser

**Table 2:** Summary of the mean distance with standard deviation (SD) and the minimum (Min) or maximum (Max) distance in mm between the STLs of the respective groups A, B, and C and the corresponding reference files for the X-, Y-, and Z-axis as well as the Euclidean distance dXYZ.

Group			Mean	SD	Min	Max
**Group A**	**ASIGA MAX UV**	dXYZ	0.12 mm	0.13 mm	0.03 mm	0.79 mm
	**DLP 1**	x	0.06 mm	0.05 mm	0.00 mm	0.21 mm
		y	0.05 mm	0.03 mm	0.01 mm	0.11 mm
		z	0.06 mm	0.14 mm	0.01 mm	0.78 mm
**Group B**	**Straumann P30+**	dXYZ	0.07 mm	0.02 mm	0.03 mm	0.10 mm
	**DLP 2**	x	0.03 mm	0.02 mm	0.00 mm	0.07 mm
		y	0.04 mm	0.02 mm	0.00 mm	0.08 mm
		z	0.03 mm	0.01 mm	0.00 mm	0.05 mm
**Group C**	**Form 3+**	dXYZ	0.26 mm	0.17 mm	0.09 mm	0.93 mm
	**SLA**	x	0.05 mm	0.05 mm	0.00 mm	0.18 mm
		y	0.17 mm	0.06 mm	0.08 mm	0.37 mm
		z	0.14 mm	0.20 mm	0.01 mm	0.91 mm

## Data Availability

The original contributions presented in this study are included in the article. Further inquiries can be directed to the corresponding author.
